# Health risks connected with ingestion of vegetables harvested from heavy metals contaminated farms in Western Nigeria

**DOI:** 10.1016/j.heliyon.2021.e07716

**Published:** 2021-08-05

**Authors:** Eguakhide Atikpo, Ehizonomhen Solomon Okonofua, Nicholas Omougbo Uwadia, Amaka Michael

**Affiliations:** a Department of Civil and Environmental Engineering, Faculty of Engineering, Delta State University, Oleh Campus, PMB 1, Delta State, Nigeria; b Department of Geomatics, University of Benin, Benin City, Nigeria; c ICT/Planning Department, National Center for Energy and Environment, University of Benin, Benin City, Nigeria; d Department of Environmental Technology, Federal University of Technology, Owerri, Imo State, Nigeria

**Keywords:** Farm soil, Vegetables, Contamination, Human health risk, Heavy metals

## Abstract

Investigation of lead (Pb), cadmium (Cd), zinc (Zn) and chromium (Cr) in soils and vegetables; and the consequent health risks connected with ingestion of the vegetables was conducted at Agbabu farm (F) settlement close to bitumen mining area of Ondo State, Nigeria. Soil and eleven vegetables were sampled from ten farms (Fs) and analyzed for Pb, Cd, Zn and Cr concentrations. Health risk parameters such as daily intake of metal (DIM), health risk index (HRI), target health quotient (THQ), and total diet target health quotient (TTHQ) were evaluated. Except Cd, other metals in soils were below their respective maximum allowable concentrations (MACs) set for agro soils. Only Cd and Pb were higher in vegetables than their respective MACs. Bioconcentration factors (BCFs) of the metals were higher in *Talinum triangulare* but lower for Cd, Zn, Cr and Pb in *Solanum macrocarpon, Vernonia amygdalina*, *Ocimum gratissimum*, and *Taraxacum officinale* respectively. DIMs of Cd, Zn and Pb for adults and children were higher than 0.0035, 0.001 and 0.300 mg/kg/day respective values of oral reference doses (RfD). The DIMs of Cr ingestion by children were above the RfD of Cr for all vegetables, while DIMs of ingesting Cr by adults were above Cr RfD for some vegetables. The (HRI >1) for Pb, Cd and Zn; (THQ >1) for Pb, Cd; and (TTHQ >1) indicated health risks in connection with the ingestion of these vegetables. These health risks were higher for children.

## 1 Introduction

Agriculture is an economy boosting activity in many countries particularly in developing countries [1] because it is a means of food production. Production of food crops particularly vegetable crops are very sensitive to soil contamination with heavy metals (HMs) mainly due to the higher risk of metal uptake and accumulation in human tissues [2, 3, 4]. Another important source of economy boom is availability of natural resources, but mining activities connected with these resources are usually located around farmlands and affect the plants [5]. Haddaway et al. in [6] reported that unchecked mining activities are a potential health risk and hazard to ecosystems [6] because mining is a source of mine spoils and disintegrated rocks wastes which are sources of HM rich effluent in the immediate environment [7, 8]. This has led to the degradation of soils [9] and poisoned crops having capabilities to trigger potential health risks if used for diets [10]. Study indicates that mine sites exhibited HMs induced deterioration rate of 60% compared with undisturbed lands [1]. Abandoned mine sites and nearby farmlands in north western Nigeria have been reported in [11] to have high pollution level with Cd, Pb, Zn, Cu and Hg, and slight pollution with Mn and Fe.

Economic development is directly connected with people health issues [12, 13, 14]. The health of people partly depends on the quality of diets they take-in [14, 15]. Vegetables are essential part of human diets [16, 17, 18]. Their ingestion has been reported [17] as the major rout of human exposure to HMs particularly when these crops are produced in polluted soils or with wastewaters [3, 19, 20]. Vegetable crops can take up various heavy metals in the soil and because they are mostly annual and consumed fresh, their health effect is higher than any other crop [21, 22, 23, 24].

Studies show that mine sites around farmlands has induced chemicals accumulation in crops' fruits and leaves - and in turn has resulted to acutely chronic health effects in humans and animals after ingesting the crops [25]. Cadmium and lead are the major heavy metals in the environment that can have toxic effects on plant and human health [26, 27].

Pains in the kidney, dizziness, miscarriages and death were reported in [28] as the consequences of ingesting HMs contaminated crops. Cadmium poses the health problems of hepatic and renal dysfunction and testicular damage [29, 30]. It has been marked for health problems associated with bone mineralization, kidney damages, and human skeletal system damages through accumulation in cells of proximal tubules [31]. Lead, as a toxicant affects all body organs [32, 33]. It reduces children's cognitive ability, mental capacity, memory loss, reproductive systems damages; and causes wrists and fingers weaknesses, memory loss, abdominal pain, and cancer of the stomach [1]. Zn is a vital nutrient for the bodies of humans [17]. However, excess of it can result to ill health manifesting as vomiting, anaemia, skin irritations, nausea, stomach cramps and metal fever with flu-like symptoms [34]. Lung cancer, asthma, kidney damage, ulcers, genetic mutations, bronchospasm, bronchitis, coughing, hepatic damage, gastric disturbances, irritation of larynx and pharynx, and weakness of immune system are health problem of exposure to Cr [35].

Because of the danger associated with ingestion of food contaminated with HMs, food protection has become a global concern [36, 37]. Therefore, it is vital to comprehend food contamination through HMs loaded vegetables harvested from HMs contaminated soils [38]. This current study is on Cd, Pb, Zn and Cr in soils and vegetables from ten Fs in Agbabu F settlement close to bitumen mining sites in Ondo State of Nigeria; and the health risks associated with ingestion of the vegetables. The study is directed towards the acquisition of information necessary for the protection of public health in pursuit of the millennium development goals.

## 2 Materials and methods

### 2.1 Study area

**Figure 1:**
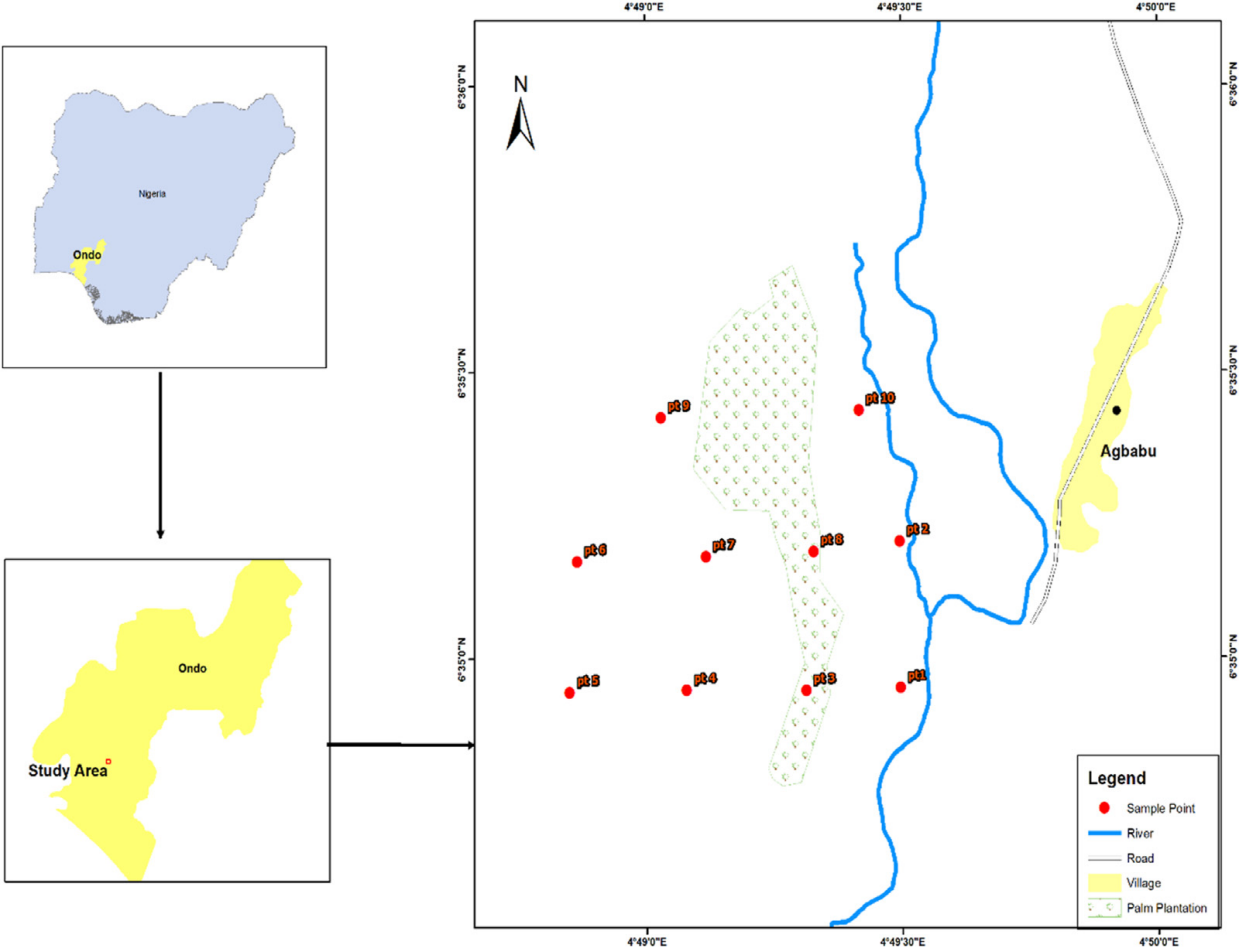
Map of study area.

Agbabu community (Figure 1) in Ondo State of Nigeria is located between latitude 6° 35′ 19″ N and longitude 4° 50′ 03″ E [39]. The community has numerous F settlements inhabited by farmers [39] cultivating different kinds of crops [40]. The community has a naturally occurring bitumen belt [39, 40] and its geology is of cretaceous tar sand bitumen deposit. According to Abatyough *et al.* in [39], 42.74 billion metric tons was the estimate of bitumen deposit in this area, making it the second largest in the globe. The climate is typically that of tropical with an average rainfall and temperature of 1837 mm and 27.1 °C [39]. The area is subjected to the two Nigerian seasons (wet and dry) [40].

### 2.2 Sample collection

From ten Fs, samples of soil were collected at 0–15 cm depth with auger. Eleven vegetables [*Cochorus olitorius (C. olitorius), Taraxacum officinale (T. officinale), Vernonia amygdalina (V. amygdalina), Amaranthus hybridus (A. hybridus), Talinum triangulare (T. triangulare), Solanum macrocarpon (S. macrocarpon), Basella alba (B. alba), Celosia argentae (C. argentae), Ocimum gratissimum (O. gratissimum), Telfairia occidentalis (T. occidentalis)* and *Crassocephalum rubens (C. rubens)*] were randomly harvested from the ten Fs. The samples were conveyed to Macgill Engineering and Technology Services, Benin City, Nigeria for acid digestion and analysis.

The samples of soil were dried with atmospheric air and sieved after grinding. Airborne deposits (dust, dirt, and other pollutants) were washed off the vegetables using distilled water [1]. After washing, the leaves of the vegetables were pluck off, dried under atmospheric air and subjected to oven drying at 65 °C for 72 h to arrive at constant weight. The oven-dried vegetable leaves were pulverized using a blender.

### 2.3 Determination of HMs

The concentrated acids (HClO_4,_ H_2_SO_4_ and HNO_3_) applied in [41] were mixed in the ratio (1:2:2). One gram of powered soil was mixed in a 250 ml flask with 15 ml of the acids mixture. The resulting mixture was kept through a night and then heated in a fume cupboard with a hotplate. Water (20 ml) was mixed with the heated mixture to cool it further. The resultant product of digestion was passed into 100 ml flask through Whatman's No. 0.45 μm paper, diluted to mark, and analyzed in triplicate for Zn, Cd, Cr and Pb using Atomic Absorption Spectrometer (UNICAM 969 model).

Adopting the technique in [1], respective pulverized vegetables (1g each) were introduced distinctly into separate pyrex beakers and mixed with HNO_3_ (10ml each). The mixture was left for 24 h at room temperature before heating it with a hotplate for 1.5 h at a temperature of 190 °C in a fume cupboard until the mixture was partially dried. The partially dried mixture was cooled before HClO_4_ (5ml) was introduced and then subjected to further heating carried out slowly until digestion was achieved. Filtration of the digested mixture was conducted with Whatman's No. 0.45 μm paper. The filtrate was collected in a flask of 100 ml capacity and diluted to flask capacity mark with distilled water. The resulting solution was kept sealed at room temperature and analyzed in triplicate to detect the concentrations of Cd, Zn, Pb and Cr using AAS (UNICAM 969 model).

### 2.4 Bioconcentration factor (BCF)

Bioconcentration factor, otherwise known as transfer or bioaccumulation factor is the ratio of HM in plant to HM in soil. It was determined by substituting the requisite data into Eq. (1) applied in [1, 17, 18, 42].

(1) $$\text{BCF}=\frac{{\text{C}}_{\text{vegetable}}}{{\text{C}}_{\text{soil}}}$$

C_vegetable_ and C_soil_ denote HM concentrations in vegetables and soil respectively.

Two ways ANOVA was conducted with Microsoft Excel 2016 at (P < 0.05) to determine significant differences in BCFs of the respective HMs for different vegetables species sourced from same F; and also for different species sourced from different Fs. In addition, to determine significant differences in BCFs of the respective HMs for same species sourced from F to F; and also for same species sourced from same F.

### 2.5 Health risk assessment

The study of DIM (daily metal intake), HRI (health risk index), THQ (target health quotient) and TTHQ (total diet THQ) was conducted to assess the health risks implication of using these vegetables as diets. The DIM was calculated using Eq. (2) applied in [18, 41, 43]; HRI was determined using Eq. (3) applied in [1, 18, 45]; THQ was determined using Eq. (4) applied in [17, 41]; and TTHQ was calculated using Eq. (5) applied in [17, 44].

(2) $$\text{DIM}=\frac{{\text{C}}_{\text{hm}}\times {\text{C}}_{\text{ft}}\times {\text{D}}_{\text{vi}}}{{\text{A}}_{\text{bw}}}$$

where C_hm_ stands for HM in vegetables. C_ft_ denotes fresh to dry weight conversion factor of vegetables. D_vi_ is daily vegetable ingestion. A_bw_ is the average body weight. A value of 0.085 in [1, 41] was substituted for C_ft_. Respective D_vi_ [187 g/p/d (gram/person/day) for adults and 130 g/p/d for children] and A_bw_ (71.3 kg for adults and 22.5 kg for children) were used.

(3) $$\text{HRI}=\frac{\text{DIM}}{\text{RfD}}$$

RfD denotes reference oral dose. The 0.001, 0.300, 0.0035 and 1.5 mg/kg/day for Cd, Zn, Pb and Cr stated in [45] and applied in [41] were inputted.

(4) $$\text{THQ}=\frac{{\text{E}}_{\text{F}}\times {\text{E}}_{\text{D}}\times {\text{F}}_{\text{IR}}\;\text{C}\times 10^{-3}}{\text{RfD}\times {\text{W}}_{\text{AB}}\times T_{A}}$$

(5) $$\text{TTHQ}=\sum_{\text{i}=1}^{\text{n}}{\left(\text{THQ}\right)}_{\text{i}}$$

E_F_ is exposure frequency (350 days/year). E_D_ is exposure duration (54 years). F_IR_ is the ingestion rate of vegetables (130 g/p/d for children and 187g/p/d for adults). RfD is 0.001, 0.300, 0.0035 and 1.5 mg/kg/day for Cd, Zn, Pb and Cr respectively. W_AB_ denotes mean body weights (22.5 kg for children and 71.3 kg for adults). T_A_ denotes non – carcinogens mean time of exposure (ED x 365 days/year). THQ >1 is an indication that intake of a particular metal through vegetable diet is of potential health risk [17, 46]; and (TTHQ) > 1 indicates negative health risk from total diets of the vegetables [17, 41].

## 3 Results and discussion

### 3.1 Zn, Cd, Pb, and Cr in soil samples

The concentrations of Zn, Cd, Pb and Cr in soils samples from all farms in Agbabu F settlement are presented as mean and standard deviation (SD) in Table 1. Pb varied in concentration from 3.91 ± 0.13 mg/kg in farm number six (F6) to 9.20 ± 0.16 mg/kg in F5 soil. Lead concentration vary in Fs soils in the order of F6 < F3 < F1 < F7 < F9 < F2 < F8 < F10 < F4 < F5. These concentration values were less than the 100 mg/kg maximum specified by standards in [47] for agro soils. Values of Pb in this study were higher than some values and lower than others reported in [48] concerning soil in Agbabu area of bitumen deposit, Nigeria. The values exceeded those reported in [41] in Challawa, Nigeria. The values were less than those reported in [1] in some regions of Pakistan; and also less than those documented in [49] in northern France.

**Table 1:** Concentration of metals in soils.

F	Metals			
	Pb	Cd	Zn	Cr
1	4.38 ± 0.12	3.52 ± 0.07	25.13 ± 1.05	17.51 ± 0.65
2	6.12 ± 0.24	4.10 ± 0.14	66.89 ± 1.01	20.79 ± 1.03
3	4.11 ± 0.18	3.21 ± 0.27	29.63 ± 0.81	22.76 ± 0.62
4	7.52 ± 0. 41	4.60 ± 0.29	101.23 ± 0.15	21.51 ± 0.93
5	9.20 ± 0.16	5.81 ± 0.12	93.18 ± 0.75	48.11 ± 0.18
6	3.91 ± 0.13	3.41 ± 0.17	35.14 ± 1.07	15.16 ± 0.13
7	4.52 ± 0.57	3.76 ± 0.13	27.48 ± 1.04	27.24 ± 0.25
8	6.56 ± 0.27	4.22 ± 0.25	54.92 ± 0.19	24.38 ± 0.17
9	5.24 ± 0.19	3.89 ± 0.16	48.61 ± 0.42	30.37 ± 1.01
10	7.12 ± 0.16	4.51 ± 0.19	90.39 ± 1.02	19.23 ± 0.21

Each value is the mean of three replicates ±SD.

The concentration of Cd varied from 3.21 ± 0.27 mg/kg in F3 to 5.81 ± 0.12 mg/kg in F5. It varied in the Fs soils in the order of F3 < F6 < F1 < F7 < F9 < F2 < F8 < F10 < F4 < F5. The concentration values in all Fs were above the maximum (3 mg/kg) set by standards in Chiroma *et al.* (2014) for agro soils. The values exceeded those reported in [48] for soil in Agbabu area of bitumen deposit, Nigeria; higher than values in [1] for selected regions agro soils in Pakistan; higher than values in [18] studied in India; and higher than values reported in [50] in China. The values were lower than some values and higher than others reported in [41] in Challawa, Nigeria.

Zinc varied in concentration from 25.13 ± 1.05 mg/kg in F1 to 101.23 ± 0.75 mg/kg in F4. The concentration varied in Fs soils in the order of F1 < F7 < F3 < F6 < F9 < F8 < F2 < F10 < F5 < F4. The concentration values in all Fs were less than the maximum allowable (300 mg/kg) for agro soils set by standards in [47]. The values were lower than some and higher than others reported in [48] about soil in Agbabu area of bitumen deposit, Nigeria. They were low compare with the ones reported in [18] in India. They exceeded the values observed in [42] in Bangladesh; and were higher than the concentration in most soils reported in [41] in Challawa, Nigeria.

Chromium varied in concentration values from 15.16 ± 0.13 mg/kg in F6 to 48.11 ± 0.18 mg/kg in F5. The concentration varied in the Fs soils in the order of F6 < F1 < F10 < F2 < F4 < F3 < F8 < F7 < F9 < F5. These concentration values were lower than some and higher than some reported in [48] concerning soil in Agbabu area of bitumen deposit, Nigeria. The values were below the ones documented in [18] in India. The values were higher in some Fs and lower in other Fs compared with the values observed in [41] in Challawa, Nigeria. The values were higher than the ones reported in [42] in Pakshi, Bangladesh.

### 3.2 Zn, Cd, Pb, and Cr in vegetables

**Figure 2:**
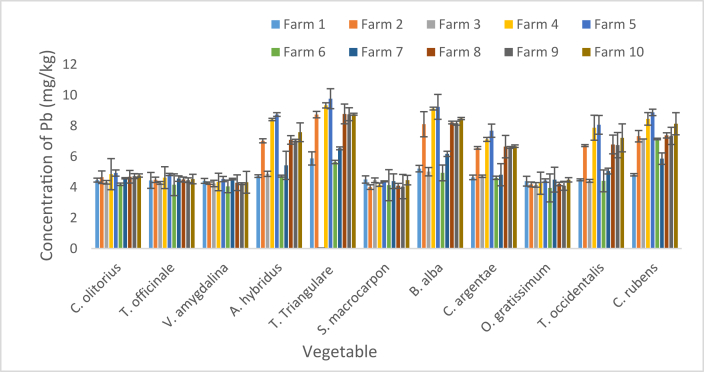
Concentration of Pb in vegetables. Bars indicates mean of three replicates ±SD.

Concentration of Pb in these vegetables from Agbabu F settlement are shown as mean and SD in Figure 2 and ranged from 3.95 ± 0.91 mg/kg in *O. gratissimum* sourced from F6 to 9.76 ± 0.65 mg/kg in *T. triangulare* from F5. In 90 % of the Fs, the minimum concentrations were recorded in *O. gratissimum* as 4.39 ± 0.32, 4.18 ± 0.16, 4.15 ± 0.15, 4.26 ± 0.72, 4.43 ± 0.11, 3.95 ± 0.91, 4.49 ± 0.81, 4.21 ± 0.11 and 4.09 ± 0.29 mg/kg in F1, F2, F3, F4, F5, F6, F7, F8 and F9 respectively. For F10, the minimum value was recorded as 4.32 ± 0.71 mg/kg in *V. amygdalina*. In all the Fs, the maximum values were recorded in *T. triangulare* as 5.87 ± 0.43, 8.73 ± 0.21, 5.81 ± 0.04, 9.33 ± 0.17, 9.76 ± 0.65, 5.64 ± 0.11, 6.53 ± 0.09, 8.77 ± 0.64, 8.75 ± 0.42 and 8.75 ± 0.06 mg/kg in F1, F2, F3, F4, F5, F6, F7, F8, F9 and F10 respectively.

**Figure 3:**
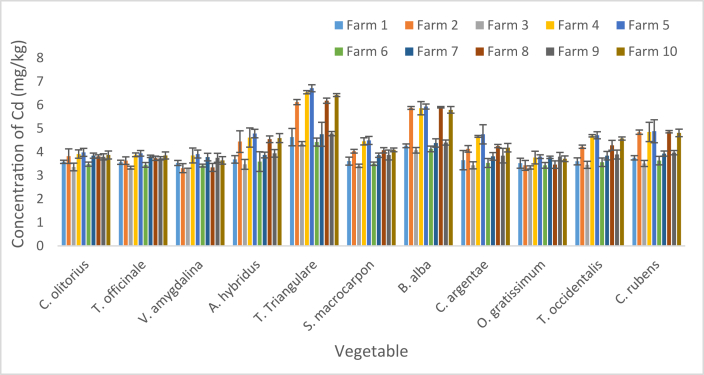
Concentration of Cd in vegetables. Bars indicates mean of three replicates ±SD.

The values of Cd concentration in the vegetables are presented as mean and SD in Figure 3. The concentration ranged from 3.31 ± 0.19 mg/kg in *V. amygdalina* sourced from F2 and 3 to 6.71 ± 0.15 mg/kg in *T. triangulare* sourced from F5. Respective minimum concentration of Cd (3.52 ± 0.21, 3.76 ± 0.27, 3.80 ± 0.08, 3.43 ± 0.12 and 3.77 ± 0.05 mg/kg) were detected in *O. gratissimum* sourced from F1, F4, F5, F6 and F7 respectively; while the respective minimum concentration (3.31 ± 0.19, 3.31 ± 0.07, 3.34 ± 0.17 and 3.64 ± 0.16 mg/kg) were detected in *V. amygdalina* sourced from F2, F3, F8 and F10 respectively. The minimum (3.73 ± 0.07 mg/kg) detected in F9 was noticed in *T. officinale*. Respective maximum concentration (4.63 ± 0.37, 6.12 ± 0.11, 4.35 ± 0.09, 6.54 ± 0.07, 6.71 ± 0.15, 4.42 ± 0.17, 4.75 ± 0.51, 6.18 ± 0.11, 4.78 ± 0.09 and 6.42 ± 0.06 mg/kg) recorded in F1, F2, F3, F4, F5, F6, F7, F8, F9 and F10 respectively were detected in *T. triangulare.* The respective Pb and Cd concentrations in all vegetables from all Fs were higher than their respective 0.3 and 0.1 mg/kg (maximum) specified by standards in [47].

The values of Pb in vegetables of this study were higher than the minimum (1.8 mg/kg) but lower than the maximum (11 mg/kg) in vegetables studied in Pakistan [1]. However, this study revealed higher Cd concentration values than the ones in vegetables reported in [1]. The respective Pb and Cd concentrations in vegetables in this study were higher than the respective concentrations recorded in vegetables studied in Pakshi, Bangladesh [42]; they were higher than those in a study conducted in India [18]; and values documented in [17] for a study conducted in Chenzhou City, China. They were also higher than the respective concentrations in some plants reported in [48] for a study conducted in Agbabu area, Nigeria. The current vegetables Pb concentrations exceeded the concentrations in vegetables studied in Challawa, Nigeria an [41]. Cd concentrations exceeded the ones in lettuce, onion and spinach but lower than the ones in tomato and Irish in the study conducted in Challawa, Nigeria and documented in [41]. In south Tehran-Iran, lower values of Cd were reported in [20] for the vegetables studied at Kahrizak. In that study, higher Pb value than the minimum in this study was reported. However, Pb values recorded in this study are generally higher than the ones recorded in Kahrizak, Tehran-Iran and documented in [20].

**Figure 4:**
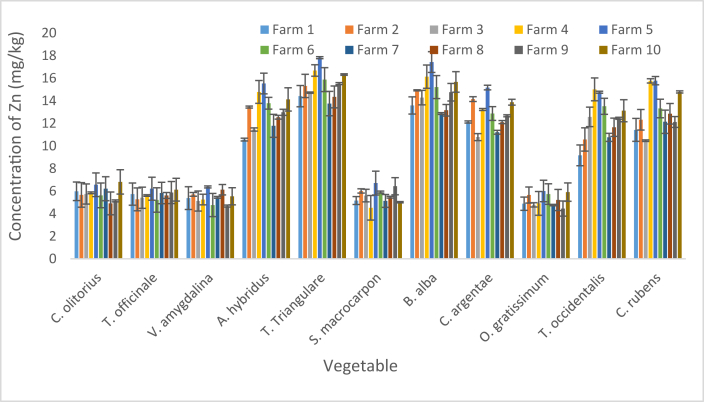
Concentration of Zn in vegetables. Bars indicates mean of three replicates ±SD.

The concentration of Zn in the vegetables are presented as mean and SD in Figure 4, and ranged from 4.45 ± 0.67 mg/kg in *O. gratissimum* sourced from F9 to 17.82 ± 0.09 mg/kg in *T. triangulare* sourced from F5 (Figure 4). The minimum values (4.88 ± 0.59, 4.78 ± 0.19, 4.91 ± 0.1.05, 6.01 ± 0.95, 4.76 ± 0.06 and 4.45 ± 0.67 mg/kg) recorded in F1, F3, F4, F5, F7 and F9 respectively were detected in *O. gratissimum*. The minimum values (4.76 ± 1.03 and 5.54 ± 0.76 mg/kg) detected in F6 and F10 respectively were observed in *V. amygdalina*, while the respective minimum (5.27 ± 1.01 and 4.91 ± 1.09 mg/kg) in F2 and F8 were detected in *T. officinale* and *C. olitorius* respectively. The maximum values (14.41 ± 0.94, 15.31 ± 1.03, 14.72 ± 0.06, 16.68 ± 0.51, 17.82 ± 0.09, 15.87 ± 1.06, 13.75 ± 1.08, 14.34 ± 0.97, 15.52 ± 0.09 and 16.32 ± 0.07 mg/kg) recorded in F1, F2, F3, F4, F5, F6, F7, F8, F9 and F10 respectively were detected in *T. triangulare*. These values were less than 100 mg/kg (maximum) specified for vegetables by standards in [47].

**Figure 5:**
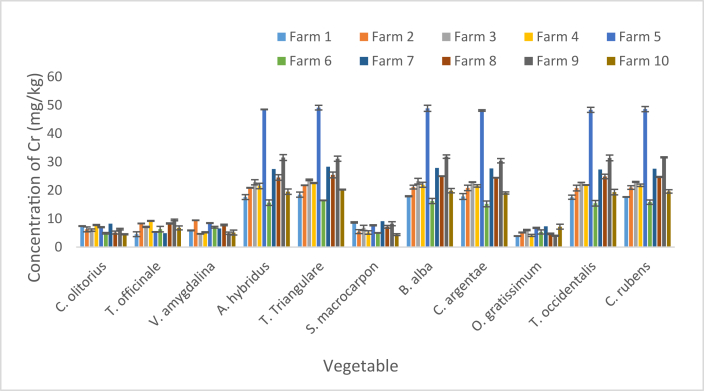
Concentration of Cr in vegetables. Bars indicates mean of three replicates ±SD.

Cr concentration in vegetables sourced from the Fs are presented as mean and SD in Figure 5. The concentration varied from 3.87 ± 0.09 mg/kg in *O. gratissimum* sourced from F1 to 49.13 ± 0.81 mg/kg in *T. triangulare* sourced from F5 (Figure 5). The respective minimum values (3.87 ± 0.09, 5.12 ± 0.13, 4.12 ± 0.36, 4.65 ± 0.23 and 3.97 ± 0.16 mg/kg) recoded in F1, F2, F4, F8 and F9 respectively were detected in *O. gratissimum*. The respective minimum values of 5.32 ± 0.09 and 4.89 ± 0.51 mg/kg recorded in F 5 and F7 respectively were detected in *T. officinale*. *V. amygdalina* bore the minimum of 4.65 ± 0.09 mg/kg in F 3; *C. olitorius* bore the minimum of 4.87 ± 0.28 mg/kg in F 6; *T. triangulare* bore the respective maximum (18.48 ± 0.91, 21.77 ± 0.08, 23.67 ± 0.26, 22.56 ± 0.15, 49.13 ± 0.81, 16.46 ± 0.08, 28.30 ± 0.78, 25.42 ± 1.01 and 20.25 ± 0.14 mg/kg) in F1, F2, F3, F4, F5, F6, F7, F8 and F10 respectively; while *B. alba* bore the maximum of 31.87 ± 0.61 mg/kg in F9.

The respective concentrations of Cr and Zn in tested vegetables exceeded the ones reported in [48] concerning some plants in Agbabu area, Nigeria; and higher than the values documented in [42] concerning a study conducted in Pakshi, Bangladesh. Zn concentration values in the studied vegetables exceeded the one reported in [17] concerning some vegetables studied at Chenzhou, China; and higher than the ones in some vegetables in Challawa, Nigeria reported in [41]. However, Zn values were below the ones documented in [18] concerning some vegetables in India. Cr values exceeded the ones documented in [18]. They were higher than the concentrations in lettuce, spinach and Irish but lower than the one in tomato studied in Challawa, Nigeria and reported in [41]. In south Tehran-Iran, lower values of Cr were reported for the vegetables studied at Kahrizak [20]. Lower values of Zn were also reported in [20] for vegetables studied at Kahrizak compared with the maximum values in this current study.

### 3.3 Bioconcentration factor (BCF)

Bioconcentration factor, otherwise known as transfer or bioaccumulation factor was determined from the ratio of the respective Zn, Cd, Cr and Pb contents of vegetables to their respective contents in soils [1, 17, 18, 42]. Been a parameter for describing the movement of metals to plant from soil, it is influenced by both vegetables and soil properties, and it forms a key reference for metals availability to persons through food chain [42].

**Figure 6:**
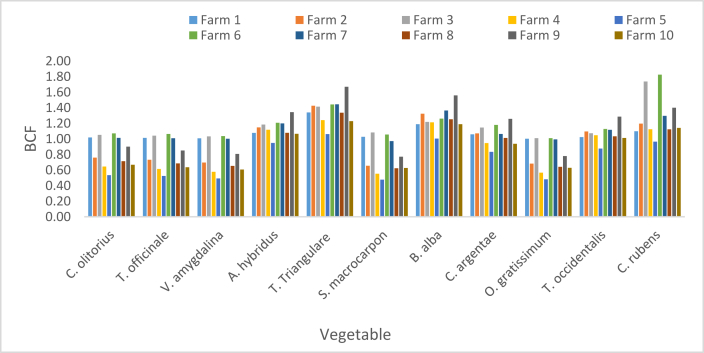
Bioconcentration factor of Pb.

The BCF of Pb presented in Figure 6 ranged from 0.48 in *S. macrocarpon* and in *O. gratissimum* sourced from F5 to 1.67 in *T. triangulare* sourced from F9. The respective BCFs of Pb in *T. triangulare* and *B. alba* sourced from all Fs were greater than one (BCF >1). Except in F5, BCFs for *A. hybridus, T. occidentalis* and *C. rubens* were greater than one in the other Fs. BCFs for *C. olitorius, T. officinale* and *V. amygdalina* were greater than one in F1, F3, F6 and F7; while the values for *S. macrocarpon* and *O. gratissium* were greater than one in F1, F3 and F6.

The respective BFC Ranges of (0–1.49) and (0.12–0.95) at Alakahia and Elemem in south-south Nigeria were reported in [51] for Pb in some vegetables. BCF above 1.11 for leafy vegetables was recorded in Chenzhou, China and reported in [17]. A range (0.005–0.073) which are values less than BCF in this study was observed in Pakshi, Bangladesh as reported [42]. Lower BCF values constituting a range (0.23–0.34) was observed in a study in Challawa, Nigeria [41]. In this study, the average BCFs revealed the bioconcentrations strengths of the studied vegetables in the order of *T. triangulare* > *C. rubens* > *B. alba* > *A. hybridus* > *T. occidentalis* > *C. argentae* > *C. olitorius* > *T. officinale* > *V. amygdalina* > *O. gratissimum* > *S. macrocarpon* (Figure 10).

**Figure 7:**
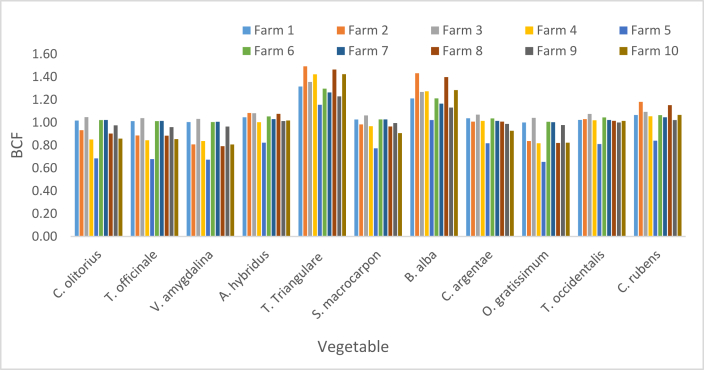
Bioconcentration factor of Cd.

BCF values of Cd presented in Figure 7 ranged from 0.65 in *O. gratissimum* harvested from F5 to 1.49 in *T. triangulare* sourced from F2. The study showed (BCFs >1) for *T. triangulare* and *B. alba* in all Fs. It showed BCFs >1 for *A. hybridus, T. occidentalis and C. rubens* in the Fs except F5. *C. olitorius, T. officinale, V. amygdalina, S. macrocarpon* and *O. gratissimum* had BCFs >1 in F1, F3, F6 and F7. *C. argentae* had BCF >1 in other farms except F5, F9 and F10. Considering the mean BCF of Cd in each vegetable harvested from the ten Fs, *T. triangulare, B. alba, C. rubens, A. hybridus and T. occidentalis* had their respective BCF >1.

Variation of the BCF was in the order of *T. triangulare* > *B. alba > C. rubens* > *A. hybridus > T. occidentalis > O. gratissimum > C. argentae > S. macrocarpon > C. olitorius > T. officinale > V. amygdalina* (Figure 10). Zero BFC of Cd in vegetables studied in Alakahia and Elemem respectively in south-south Nigeria were reported in [51]; and the study conducted in Pakshi, Bangladesh reported in [42]. BCF range (0.0000652–2.63) for some vegetables studied in Challawa, Nigeria and reported in [41] showed that only 0.0000652 is less than the BCFs of Cd in this study.

**Figure 8:**
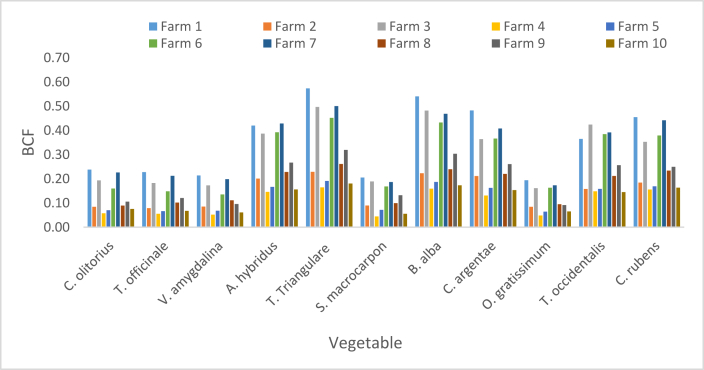
Bioconcentration factor of Zn.

Zn BCFs in vegetables were respectively less than one. The lowest value of 0.04 was recorded in *S. macrocarpon* sourced from F4 while the highest was 0.57 in *T. triangulare* sourced from F1 (Figure 8). The order of mean BCFs in the vegetables was *T. triangulare* > *B. alba > A. hybridus > C. rubens* > *C. argentae > T. occidentalis > C. olitorius > T. officinale > S. macrocarpon > V. amygdalina > O. gratissimum* (Figure 10). Zn BCFs above 1.11 was reported in [17] for leafy vegetables in Chenzhou, China. A range (0.015–0.138) of BCFs was reported in Pakshi, Bangladesh [42]; and a range (0.26–0.60) was reported in Challawa, Nigeria [41].

**Figure 9:**
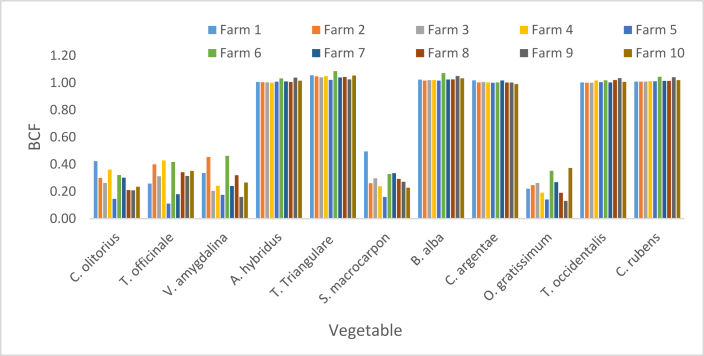
Bioconcentration factor of Cr.

BCF values of Cr presented in Figure 9 shows a minimum of 0.11 in *T. officinale* sourced from F5 and maximum value of 1.09 in *T. triangulare* sourced from F6. BCF values for *T. triangulare*, *B. alba, A. hybridus, C. rubens*, *C. argentae* and *T. occidentalis* were greater than one, while the values for *C. olitorius, T. officinale, S. macrocarpon, V. amygdalina* and *O. gratissimum* were lower than one. The BCFs of Cr in this study were greater than the ones recorded in Pakshi, Bangladesh [42]; greater than the ones in onion and Irish but less than the ones in tomato, lettuce and spinach studied in Challawa, Nigeria [41]. Respective Ranges of (0–0.14) and (0.05–0.21) were reported in [51] for vegetables in Alakahia and Elemem respectively in south-south Nigeria.

**Figure 10:**
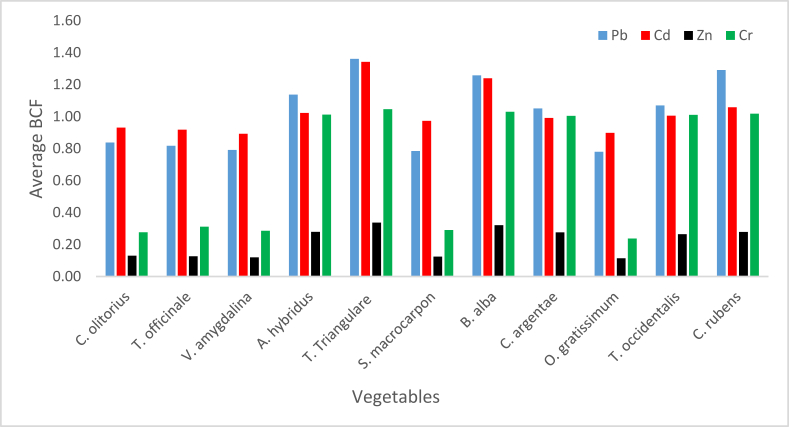
Average bioconcentration factor.

The respective average BCFs of Zn, Cd, Cr and Pb are presented in Figure 10 in the order of Pb > Cd > Cr > Zn. High BCF implies great potency of plant to attract metals from soil, or poor capability of soils to retain metal [42]; while low BCF implies high biding ability of metals to soil [52].

ANOVA portrayed significant differences in values of BCF for Pb, Cd, Zn and Cr respectively between different species in same F; and in different Fs also. It also portrayed significant differences in same species sourced from different Fs; and sourced from same F also. These significant differences are in line with the findings that both vegetables and soil properties influence BCF [42]. Alloway and Ayres in [53] mentioned that metals mobility from soils to plants depends on soil characteristics (physical and chemical); and vegetable species.

### 3.4 Health risk

Intake of metals even at low concentrations is of adverse health consequences which become obvious after exposure for many years [54]. Farm produce from Agbabu F settlement is critical to the nutrition of the people in the community and beyond. The vegetables harvested from the studied Fs are sold in the community's and other communities' markets. They are also transported to markets outside Ondo State. For this purpose, it became imperative to look into the health risks attached to the vegetables ingestion from Agbabu F settlement. To achieve this, certain parameters (HRI, DIM, THQ and TTHQ) were computed to determine the health risks connected with vegetables ingestion from these Fs.

In Table 2, the minimum DIM of Pb recorded for adults was 0.88 mg/kg/day, while the minimum for children was 1.94 mg/kg/day. These values were observed in *O. gratissimum* sourced from F 6. The maximum DIM of Pb for adults was 2.18 mg/kg/day, while the maximum for children was 4.79 mg/kg/day. These values were discovered in *T. triangulare* sourced from F5. These values exceeded the RfD of Pb referred in [17, 18, 41]. The values were greater than the values for selected vegetables in Pakistan [1]; higher than the ones discovered in south-south, Nigeria [51]; higher than the values recoded in Zhejiang, China [28]; higher than the values recoded for lettuce, tomato and onion in Nigeria [41]. DIM range (0.26–2.00 mg/kg/day) was discovered for some vegetables in India [18].

**Table 2:** Daily intake of metals.

F	HM	Individual	Vegetable/DIM										
			*C. olitorius*	*T. officinale*	*V. amygdalina*	*A. hybridus*	*T. Triangulare*	*S. macrocarpon*	*B. alba*	*C. argentae*	*O. gratissimum*	*T. occidentalis*	*C. rubens*
			DIM	DIM	DIM	DIM	DIM	DIM	DIM	DIM	DIM	DIM	DIM
F 1	Cd	Adults	0.80	0.79	0.79	0.82	1.03	0.80	0.95	0.81	0.78	0.80	0.84
		Children	1.76	1.75	1.73	1.81	2.27	1.77	2.09	1.79	1.73	1.77	1.84
	Pb	Adults	0.99	0.99	0.98	1.05	1.31	1.00	1.16	1.03	0.98	1.00	1.07
		Children	2.19	2.18	2.17	2.32	2.88	2.21	2.56	2.28	2.16	2.20	2.36
	Zn	Adults	1.33	1.28	1.20	2.35	3.21	1.15	3.03	2.70	1.09	2.04	2.55
		Children	2.94	2.81	2.64	5.19	7.08	2.53	6.67	5.95	2.40	4.50	5.61
	Cr	Adults	1.65	1.01	1.31	3.93	4.12	1.93	3.99	3.97	0.86	3.91	3.94
		Children	3.64	2.22	2.88	8.65	9.08	4.26	8.80	8.75	1.90	8.62	8.68
F 2	Cd	Adults	0.85	0.81	0.74	0.99	1.36	0.90	1.31	0.92	0.76	0.94	1.08
		Children	1.88	1.78	1.63	2.18	3.01	1.98	2.88	2.03	1.68	2.07	2.38
	Pb	Adults	1.04	1.00	0.95	1.56	1.95	0.89	1.81	1.46	0.93	1.50	1.63
		Children	2.28	2.20	2.09	3.45	4.29	1.97	3.98	3.22	2.05	3.30	3.59
	Zn	Adults	1.26	1.17	1.27	3.00	3.41	1.34	3.33	3.15	1.26	2.36	2.75
		Children	2.78	2.59	2.80	6.60	7.52	2.95	7.33	6.95	2.77	5.19	6.05
	Cr	Adults	1.39	1.85	2.10	4.65	4.85	1.21	4.71	4.65	1.14	4.63	4.68
		Children	3.06	4.09	4.63	10.25	10.69	2.67	10.37	10.23	2.51	10.21	10.30
F 3	Cd	Adults	0.75	0.74	0.74	0.77	0.97	0.76	0.91	0.76	0.74	0.77	0.78
		Children	1.65	1.64	1.63	1.70	2.14	1.67	2.00	1.68	1.64	1.69	1.72
	Pb	Adults	0.96	0.95	0.95	1.09	1.30	0.99	1.12	1.05	0.93	0.98	1.59
		Children	2.12	2.10	2.08	2.39	2.85	2.19	2.46	2.31	2.04	2.17	3.51
	Zn	Adults	1.28	1.21	1.14	2.55	3.28	1.25	3.18	2.40	1.07	2.80	2.33
		Children	2.82	2.66	2.51	5.62	7.23	2.75	7.01	5.29	2.35	6.17	5.14
	Cr	Adults	1.33	1.59	1.04	5.09	5.28	1.51	5.17	5.10	1.33	5.08	5.12
		Children	2.94	3.49	2.28	11.21	11.62	3.32	11.40	11.24	2.94	11.18	11.28
F 4	Cd	Adults	0.87	0.86	0.86	1.03	1.46	0.99	1.31	1.04	0.84	1.05	1.08
		Children	1.92	1.91	1.89	2.26	3.21	2.19	2.88	2.29	1.85	2.30	2.38
	Pb	Adults	1.08	1.03	0.97	1.87	2.08	0.93	2.03	1.59	0.95	1.75	1.88
		Children	2.38	2.27	2.13	4.13	4.58	2.04	4.48	3.49	2.09	3.87	4.15
	Zn	Adults	1.30	1.25	1.17	3.29	3.72	1.01	3.60	2.95	1.09	3.35	3.51
		Children	2.87	2.76	2.58	7.26	8.19	2.22	7.92	6.50	2.41	7.37	7.74
	Cr	Adults	1.73	2.05	1.16	4.80	5.03	1.14	4.89	4.81	0.92	4.88	4.85
		Children	3.81	4.52	2.55	10.56	11.08	2.52	10.77	10.59	2.02	10.74	10.69
F 5	Cd	Adults	0.89	0.88	0.87	1.07	1.50	1.00	1.32	1.06	0.85	1.05	1.09
		Children	1.95	1.93	1.92	2.35	3.30	2.21	2.91	2.33	1.87	2.31	2.40
	Pb	Adults	1.10	1.08	1.01	1.95	2.18	0.98	2.06	1.71	0.99	1.80	1.98
		Children	2.42	2.37	2.22	4.29	4.79	2.15	4.53	3.77	2.18	3.96	4.36
	Zn	Adults	1.46	1.38	1.42	3.46	3.97	1.50	3.89	3.38	1.34	3.29	3.52
		Children	3.22	3.05	3.13	7.63	8.75	3.30	8.56	7.45	2.95	7.25	7.75
	Cr	Adults	1.56	1.19	1.88	10.81	10.95	1.71	10.90	10.73	1.51	10.78	10.84
		Children	3.44	2.61	4.15	23.82	24.13	3.77	23.81	23.63	3.33	23.75	23.88
F 6	Cd	Adults	0.78	0.77	0.76	0.80	0.99	0.78	0.92	0.79	0.76	0.79	0.81
		Children	1.71	1.69	1.68	1.76	2.17	1.72	2.03	1.73	1.68	1.75	1.78
	Pb	Adults	0.93	0.93	0.90	1.05	1.26	0.92	1.10	1.03	0.88	0.98	1.59
		Children	2.06	2.04	1.99	2.32	2.77	2.03	2.42	2.26	1.94	2.17	3.51
	Zn	Adults	1.25	1.16	1.06	3.07	3.54	1.32	3.39	2.87	1.28	3.01	2.97
		Children	2.76	2.56	2.34	6.77	7.79	2.90	7.47	6.32	2.81	6.63	6.54
	Cr	Adults	1.09	1.41	1.56	3.49	3.67	1.11	3.62	3.39	1.19	3.44	3.53
		Children	2.39	3.10	3.44	7.69	8.08	2.45	7.98	7.47	2.62	7.58	7.77
F 7	Cd	Adults	0.86	0.85	0.84	0.86	1.06	0.86	0.98	0.85	0.84	0.86	0.88
		Children	1.89	1.87	1.86	1.90	2.33	1.90	2.15	1.87	1.85	1.89	1.93
	Pb	Adults	1.02	1.02	1.01	1.21	1.46	0.98	1.38	1.07	1.00	1.13	1.31
		Children	2.25	2.24	2.22	2.66	3.21	2.16	3.03	2.36	2.21	2.48	2.88
	Zn	Adults	1.38	1.30	1.21	2.63	3.07	1.14	2.87	2.50	1.06	2.40	2.71
		Children	3.05	2.86	2.68	5.79	6.75	2.52	6.32	5.51	2.34	5.28	5.96
	Cr	Adults	1.83	1.09	1.46	6.13	6.31	2.03	6.22	6.18	1.63	6.09	6.16
		Children	4.04	2.40	3.22	13.51	13.90	4.47	13.70	13.60	3.59	13.41	13.56
F 8	Cd	Adults	0.85	0.83	0.74	1.01	1.38	0.91	1.32	0.95	0.77	0.95	1.08
		Children	1.87	1.83	1.64	2.23	3.04	2.00	2.90	2.09	1.70	2.10	2.39
	Pb	Adults	1.04	1.00	0.96	1.58	1.96	0.91	1.83	1.48	0.94	1.51	1.64
		Children	2.30	2.21	2.11	3.48	4.31	2.01	4.03	3.26	2.07	3.33	3.62
	Zn	Adults	1.09	1.25	1.36	2.80	3.20	1.22	2.93	2.70	1.16	2.60	2.86
		Children	2.41	2.76	3.01	6.16	7.04	2.68	6.46	5.95	2.56	5.72	6.31
	Cr	Adults	1.14	1.85	1.74	5.47	5.67	1.59	5.57	5.44	1.04	5.54	5.51
		Children	2.51	4.09	3.83	12.04	12.48	3.51	12.27	11.99	2.28	12.21	12.14
F 9	Cd	Adults	0.84	0.83	0.84	0.88	1.07	0.86	0.98	0.86	0.85	0.87	0.89
		Children	1.86	1.83	1.84	1.93	2.35	1.90	2.16	1.89	1.87	1.91	1.95
	Pb	Adults	1.05	0.99	0.94	1.57	1.95	0.90	1.82	1.47	0.91	1.50	1.64
		Children	2.32	2.19	2.08	3.46	4.30	1.98	4.01	3.24	2.01	3.31	3.60
	Zn	Adults	1.14	1.31	1.04	2.89	3.46	1.43	3.29	2.82	0.99	2.78	2.70
		Children	2.52	2.88	2.29	6.37	7.62	3.16	7.25	6.22	2.19	6.11	5.95
	Cr	Adults	1.41	2.13	1.08	7.03	6.94	1.83	7.10	6.78	0.89	7.00	7.05
		Children	3.10	4.69	2.39	15.49	15.28	4.04	15.65	14.94	1.95	15.43	15.52
F 10	Cd	Adults	0.86	0.86	0.81	1.02	1.43	0.91	1.29	0.93	0.83	1.02	1.07
		Children	1.90	1.89	1.79	2.25	3.15	2.01	2.84	2.05	1.82	2.24	2.36
	Pb	Adults	1.06	1.01	0.96	1.69	1.95	0.99	1.89	1.49	1.00	1.61	1.81
		Children	2.34	2.22	2.12	3.72	4.30	2.19	4.16	3.28	2.20	3.54	3.99
	Zn	Adults	1.52	1.36	1.24	3.15	3.64	1.12	3.49	3.09	1.32	2.92	3.29
		Children	3.34	3.01	2.72	6.93	8.01	2.46	7.70	6.81	2.90	6.44	7.26
	Cr	Adults	1.01	1.51	1.14	4.35	4.51	0.98	4.43	4.25	1.60	4.32	4.37
		Children	2.21	3.32	2.51	9.59	9.95	2.15	9.75	9.36	3.52	9.51	9.63

The minimum Cd intake by adults and children were 0.74 and 1.63 mg/kd/day respectively through the ingestion of *V. amygdalina* sourced from F1 and F2 respectively; while the maximum intakes were 1.50 and 3.30 mg/kg/day respectively through diet of *T. triangulare* sourced from F5 (Table 2). These values exceed RfD of Cd documented in [17, 18, 41]. DIMs in this study exceeded the DIMs recorded in a study conducted in Pakistan [1]; higher than the ones recorded for some vegetables in Challawa, Nigeria [41]; higher than the values recorded in a study conducted in south-south, Nigeria [51]; and higher than DIMs recorded in Zhejiang, China [28]. DIM range of 0.11–0.67 mg/kg/day for some selected vegetables studied in India were reported in [18].

Zn minimum DIM for adults was 0.99 mg/kg/day and the minimum for children was 2.19 mg/kg/day. These were connected with *O. gratissimum* sourced from F9. The maximum DIM value for adults was 3.64 mg/kg/day and the maximum for children was 8.01 mg/kg/day. These were in connection with *T triangulare* sourced from F10 (Table 2). These values were higher than Zn RfD referred in [17, 18, 41]. These Zn DIMs exceeded Zn DIMs for some vegetables in India [18]. Respective DIMs of 6.14, 13.9 and 11.7 mg/kg/day for lettuce, tomato and onion were reported in Challawa, Nigeria [41].

The minimum Cr DIM for adults was 0.86 mg/kg/day and the minimum for children was 1.90 mg/kg/day. These values were in connection with ingestion of *O. gratissimum* sourced from F1. The maximum DIMs for adults and children were 10.95 and 24.13 mg/kg/day respectively through diet of *T triangulare* from F5 (Table 2). Cr DIM values for all vegetables intake by children from all Fs were greater than the RfD of Cr. For adults, Cr DIM were greater than the RfD for vegetables from F5 and other Fs except for *T. Officinale* from F1, F6 and F7; *V. amygdalina* from F1, F3, F4, F7, F9 and F10; *O. gratissimum* from F1, F2, F3, F4, F6, F8 and F9. This applied to *C. olitorius* from F2, F3, F6, F8, F9 and F10; and *S. macrocarpon* from F4, F6 and F10. Cr DIM range of 0.4–7.2 mg/kg/day was documented in [18] for some vegetables in India. Respective Cr DIMs of 0.14, 7.23 and 0.03 mg/kg/day for lettuce, tomato and onion for adults and 0.004, 0.19 and 0.007 mg/kg/day for children were recorded in Challawa, Nigeria [41]. The DIMs in this study are higher than DIMs recorded in a study conducted in Tamale, Gnana [19]; higher than DIMs determined in south-south, Nigeria [51]; and higher than DIMs in a study conducted in Zhejiang, China [28].

The HRIs are shown in Table 3. Their respective values followed the trend of the respective DIMs presented in Table 2. The HRI >1 in the cases of Cd, Zn and Pb. For Cr, HRI >1 except in the previously mentioned cases were the adults DIMs of Cr were less than Cr RfD. The (HRI >1) points at risks in relation to the affected vegetables from the F settlement. The risks magnitude was in the order of Cd > Pb > Zn > Cr showing Cd as likely to constitute the greatest health risk while Cr was likely to constitute the least health risk. HRI >1 was also reported in [41] for Cd, Pb, Zn and Cr in some selected vegetables except in lettuce. HRI <1 was reported in [51] for Pb, Cr and Cd in vegetables in south-south, Nigeria; and also reported in [28] for Pb, Cr and Cd in vegetables in Zhejiang, China. Rehman *et al.* in [1] reported HRI <1 for Pb in most species of vegetables but HRI >1 for some of the vegetables consumed by children. They reported Cd HRI range of 0.1–1.3 for adults and 0.002 to 1.9 for children. Ramteke *et al.* in [18] reported HRI of these metals in the order of Cd > Pb > Zn > Cr with HRI >1 in all the vegetables except for Cr with HRI <1 for *C. sativum, S. melongena and S. lycopersicum.*

**Table 3:** Health risk index.

F	HM	Individual	Vegetable/HRI										
			*C. olitorius*	*T. officinale*	*V. amygdalina*	*A. hybridus*	*T. Triangulare*	*S. macrocarpon*	*B. alba*	*C. argentae*	*O. gratissimum*	*T. occidentalis*	*C. rubens*
			HRI	HRI	HRI	HRI	HRI	HRI	HRI	HRI	HRI	HRI	HRI
F 1	Cd	Adults	798.09	793.64	786.95	820.39	1032.17	804.78	949.69	813.70	784.72	802.55	835.99
		Children	1758.18	1748.36	1733.62	1807.29	2273.84	1772.91	2092.13	1792.56	1728.71	1768.00	1841.67
	Pb	Adults	284.08	282.80	280.89	300.64	373.89	286.63	331.85	295.54	279.62	285.35	306.37
		Children	625.82	623.01	618.80	662.30	823.66	631.43	731.05	651.07	615.99	628.62	674.93
	Zn	Adults	4.44	4.26	4.00	7.85	10.71	3.83	10.09	9.01	3.63	6.81	8.49
		Children	9.79	9.38	8.81	17.29	23.59	8.45	22.23	19.84	7.99	15.00	18.69
	Cr	Adults	1.10	0.67	0.87	2.62	2.75	1.29	2.66	2.65	0.58	2.61	2.63
		Children	2.43	1.48	1.92	5.77	6.05	2.84	5.87	5.83	1.27	5.75	5.79
F 2	Cd	Adults	851.60	809.24	737.90	989.81	1364.34	898.41	1308.61	920.71	764.65	940.77	1078.99
		Children	1876.04	1782.73	1625.58	2180.53	3005.60	1979.18	2882.82	2028.29	1684.51	2072.49	2376.98
	Pb	Adults	296.18	285.35	271.34	447.14	556.05	255.42	515.93	417.84	266.24	427.39	466.24
		Children	652.48	628.62	597.75	985.03	1224.97	562.67	1136.57	920.48	586.53	941.53	1027.12
	Zn	Adults	4.21	3.92	4.24	9.99	11.38	4.47	11.09	10.51	4.20	7.85	9.16
		Children	9.27	8.63	9.35	22.00	25.06	9.84	24.42	23.16	9.25	17.30	20.17
	Cr	Adults	0.93	1.24	1.40	3.10	3.24	0.81	3.14	3.10	0.76	3.09	3.12
		Children	2.04	2.72	3.09	6.84	7.13	1.78	6.91	6.82	1.68	6.81	6.87
F 3	Cd	Adults	749.05	742.36	737.90	773.57	969.75	760.20	907.33	764.65	744.59	769.11	782.49
		Children	1650.13	1635.40	1625.58	1704.16	2136.33	1674.69	1998.82	1684.51	1640.31	1694.33	1723.80
	Pb	Adults	275.16	272.61	270.07	310.19	370.07	283.44	319.11	300.00	264.33	280.89	454.78
		Children	606.17	600.56	594.95	683.35	815.24	624.41	702.99	660.90	582.32	618.80	1001.87
	Zn	Adults	4.27	4.02	3.80	8.51	10.94	4.16	10.61	8.01	3.55	9.34	7.77
		Children	9.40	8.86	8.38	18.74	24.10	9.17	23.38	17.65	7.83	20.58	17.12
	Cr	Adults	0.89	1.06	0.69	3.39	3.52	1.00	3.45	3.40	0.89	3.38	3.41
		Children	1.96	2.33	1.52	7.47	7.75	2.21	7.60	7.49	1.96	7.46	7.52
F 4	Cd	Adults	871.66	864.97	858.29	1027.71	1457.97	992.04	1306.38	1038.86	838.22	1045.55	1081.22
		Children	1920.24	1905.51	1890.78	2264.02	3211.87	2185.44	2877.91	2288.58	1846.58	2303.31	2381.89
	Pb	Adults	308.92	294.27	276.43	535.67	594.27	264.97	580.90	452.87	271.34	501.28	538.22
		Children	680.54	648.27	608.98	1180.07	1309.16	583.72	1279.70	997.66	597.75	1104.30	1185.68
	Zn	Adults	4.35	4.17	3.90	10.98	12.39	3.36	11.99	9.83	3.65	11.15	11.71
		Children	9.58	9.18	8.59	24.20	27.31	7.40	26.41	21.66	8.04	24.57	25.80
	Cr	Adults	1.15	1.37	0.77	3.20	3.35	0.76	3.26	3.20	0.61	3.25	3.23
		Children	2.54	3.02	1.70	7.04	7.39	1.68	7.18	7.06	1.35	7.16	7.12
F 5	Cd	Adults	887.27	878.35	871.66	1065.61	1495.87	1000.96	1321.98	1058.92	847.14	1050.01	1087.90
		Children	1954.62	1934.98	1920.24	2347.51	3295.36	2205.09	2912.29	2332.78	1866.22	2313.13	2396.62
	Pb	Adults	313.38	307.65	288.54	556.05	621.66	278.98	587.90	489.17	282.17	513.38	564.97
		Children	690.36	677.73	635.64	1224.97	1369.50	614.59	1295.13	1077.64	621.61	1130.96	1244.62
	Zn	Adults	4.87	4.61	4.73	11.54	13.24	4.99	12.95	11.27	4.47	10.97	11.73
		Children	10.74	10.17	10.43	25.42	29.17	10.98	28.53	24.83	9.84	24.16	25.83
	Cr	Adults	1.04	0.79	1.26	7.21	7.30	1.14	7.27	7.15	1.01	7.19	7.23
		Children	2.30	1.74	2.77	15.88	16.09	2.51	15.88	15.75	2.22	15.83	15.92
F 6	Cd	Adults	775.80	769.11	762.42	800.32	985.36	780.26	920.71	786.95	764.65	793.64	809.24
		Children	1709.07	1694.33	1679.60	1763.09	2170.71	1718.89	2028.29	1733.62	1684.51	1748.36	1782.73
	Pb	Adults	266.88	264.97	257.96	300.64	359.24	263.06	314.01	293.63	251.59	280.89	454.78
		Children	587.93	583.72	568.29	662.30	791.39	579.51	691.77	646.86	554.25	618.80	1001.87
	Zn	Adults	4.18	3.87	3.54	10.24	11.79	4.39	11.30	9.56	4.26	10.04	9.90
		Children	9.20	8.53	7.79	22.56	25.98	9.67	24.90	21.07	9.38	22.12	21.81
	Cr	Adults	0.72	0.94	1.04	2.33	2.45	0.74	2.41	2.26	0.79	2.29	2.35
		Children	1.59	2.07	2.30	5.12	5.39	1.63	5.32	4.98	1.75	5.06	5.18
F 7	Cd	Adults	856.06	849.37	842.68	862.74	1058.92	860.51	976.44	849.37	840.45	856.06	876.12
		Children	1885.87	1871.13	1856.40	1900.60	2332.78	1895.69	2151.07	1871.13	1851.49	1885.87	1930.07
	Pb	Adults	291.72	290.45	288.54	345.23	415.93	279.62	393.00	306.37	285.99	321.66	373.25
		Children	642.65	639.85	635.64	760.52	916.27	615.99	865.76	674.93	630.03	708.60	822.26
	Zn	Adults	4.61	4.33	4.05	8.75	10.22	3.81	9.56	8.33	3.54	8.00	9.02
		Children	10.17	9.54	8.92	19.28	22.51	8.40	21.07	18.35	7.79	17.61	19.87
	Cr	Adults	1.22	0.73	0.97	4.09	4.21	1.35	4.15	4.12	1.09	4.06	4.10
		Children	2.69	1.60	2.15	9.00	9.27	2.98	9.13	9.07	2.40	8.94	9.04
F 8	Cd	Adults	849.37	831.53	744.59	1012.11	1377.72	907.33	1315.29	947.46	771.34	954.15	1083.45
		Children	1871.13	1831.84	1640.31	2229.64	3035.07	1998.82	2897.56	2087.22	1699.24	2101.96	2386.80
	Pb	Adults	298.09	286.63	273.25	450.96	558.60	260.51	522.93	422.93	268.15	431.85	469.43
		Children	656.69	631.43	601.96	993.45	1230.58	573.90	1152.01	931.71	590.74	951.35	1034.14
	Zn	Adults	3.65	4.17	4.55	9.32	10.66	4.06	9.78	9.00	3.87	8.66	9.55
		Children	8.04	9.18	10.02	20.53	23.48	8.94	21.54	19.82	8.53	19.07	21.04
	Cr	Adults	0.76	1.24	1.16	3.64	3.78	1.06	3.71	3.63	0.69	3.70	3.67
		Children	1.68	2.72	2.55	8.03	8.32	2.34	8.18	7.99	1.52	8.14	8.09
F 9	Cd	Adults	844.91	831.53	835.99	878.35	1065.61	862.74	980.90	856.06	847.14	867.20	885.04
		Children	1861.31	1831.84	1841.67	1934.98	2347.51	1900.60	2160.89	1885.87	1866.22	1910.42	1949.71
	Pb	Adults	300.64	284.08	269.43	448.41	557.33	257.33	520.39	419.75	260.51	429.30	467.52
		Children	662.30	625.82	593.54	987.83	1227.78	566.88	1146.39	924.69	573.90	945.74	1029.93
	Zn	Adults	3.81	4.36	3.47	9.65	11.53	4.78	10.97	9.42	3.31	9.25	9.01
		Children	8.40	9.61	7.64	21.25	25.41	10.53	24.16	20.74	7.28	20.38	19.84
	Cr	Adults	0.94	1.42	0.72	4.69	4.63	1.22	4.74	4.52	0.59	4.67	4.70
		Children	2.07	3.12	1.59	10.33	10.19	2.69	10.43	9.96	1.30	10.28	10.35
F 10	Cd	Adults	862.74	858.29	811.47	1023.25	1431.22	911.79	1290.77	931.85	827.08	1018.80	1072.30
		Children	1900.60	1890.78	1787.64	2254.20	3152.93	2008.64	2843.53	2052.84	1822.02	2244.38	2362.24
	Pb	Adults	303.19	288.54	275.16	482.81	557.33	284.08	539.49	424.84	285.35	459.24	517.84
		Children	667.91	635.64	606.17	1063.61	1227.78	625.82	1188.49	935.92	628.62	1011.69	1140.78
	Zn	Adults	5.06	4.55	4.12	10.49	12.13	3.72	11.64	10.31	4.39	9.75	10.98
		Children	11.15	10.02	9.07	23.11	26.72	8.20	25.65	22.71	9.67	21.48	24.20
	Cr	Adults	0.67	1.00	0.76	2.90	3.01	0.65	2.95	2.83	1.07	2.88	2.91
		Children	1.48	2.21	1.68	6.39	6.63	1.43	6.50	6.24	2.35	6.34	6.42

The HRIs for children were generally higher. These are signals of greater risks for children. Rehman et al. in [1] and Edogbo et al. in [41] also reported higher HRI values for children. Variation in HRI of children and adults is due to HRI dependence on daily vegetable intake and persons' body weights which differ between adults and children [1, 18, 41].

The occurrence of health effect of non-carcinogenic nature with years was also assessed with the THQ of the metals. The THQs of the metals were calculated and presented in Table 4. The principles of usage of THQ is that if THQ >1, the ingestion of such metal is likely to cause health problems with time [17, 41].

**Table 4:** Target health quotient.

F	HM	Individual	Vegetable/THQ										
			*C. olitorius*	*T. officinale*	*V. amygdalina*	*A. hybridus*	*T. Triangulare*	*S. macrocarpon*	*B. alba*	*C. argentae*	*O. gratissimum*	*T. occidentalis*	*C. rubens*
			THQ	THQ	THQ	THQ	THQ	THQ	THQ	THQ	THQ	THQ	THQ
F 1	Cd	Adults	9.00	8.95	8.88	9.25	11.64	9.08	10.71	9.18	8.85	9.05	9.43
		Children	19.83	19.72	19.56	20.39	25.65	20.00	23.60	20.22	19.50	19.95	20.78
	Pb	Adults	3.20	3.19	3.17	3.39	4.22	3.23	3.74	3.33	3.15	3.22	3.46
		Children	7.06	7.03	6.98	7.47	9.29	7.12	8.25	7.34	6.95	7.09	7.61
	Zn	Adults	0.05	0.05	0.05	0.09	0.12	0.04	0.11	0.10	0.04	0.08	0.10
		Children	0.11	0.11	0.10	0.20	0.27	0.10	0.25	0.22	0.09	0.17	0.21
	Cr	Adults	0.01	0.01	0.01	0.03	0.03	0.01	0.03	0.03	0.01	0.03	0.03
		Children	0.03	0.02	0.02	0.07	0.07	0.03	0.07	0.07	0.01	0.06	0.07
F 2	Cd	Adults	9.61	9.13	8.32	11.17	15.39	10.14	14.76	10.39	8.63	10.61	12.17
		Children	21.16	20.11	18.34	24.60	33.91	22.33	32.52	22.88	19.00	23.38	26.82
	Pb	Adults	3.34	3.22	3.06	5.04	6.27	2.88	5.82	4.71	3.00	4.82	5.26
		Children	7.36	7.09	6.74	11.11	13.82	6.35	12.82	10.38	6.62	10.62	11.59
	Zn	Adults	0.05	0.04	0.05	0.11	0.13	0.05	0.13	0.12	0.05	0.09	0.10
		Children	0.10	0.10	0.11	0.25	0.28	0.11	0.28	0.26	0.10	0.20	0.23
	Cr	Adults	0.01	0.01	0.02	0.04	0.04	0.01	0.04	0.03	0.01	0.03	0.04
		Children	0.02	0.03	0.03	0.08	0.08	0.02	0.08	0.08	0.02	0.08	0.08
F 3	Cd	Adults	8.45	8.37	8.32	8.73	10.94	8.58	10.24	8.63	8.40	8.68	8.83
		Children	18.62	18.45	18.34	19.22	24.10	18.89	22.55	19.00	18.50	19.11	19.45
	Pb	Adults	3.10	3.08	3.05	3.50	4.17	3.20	3.60	3.38	2.98	3.17	5.13
		Children	6.84	6.78	6.71	7.71	9.20	7.04	7.93	7.46	6.57	6.98	11.30
	Zn	Adults	0.05	0.05	0.04	0.10	0.12	0.05	0.12	0.09	0.04	0.11	0.09
		Children	0.11	0.10	0.09	0.21	0.27	0.10	0.26	0.20	0.09	0.23	0.19
	Cr	Adults	0.01	0.01	0.01	0.04	0.04	0.01	0.04	0.04	0.01	0.04	0.04
		Children	0.02	0.03	0.02	0.08	0.09	0.02	0.09	0.08	0.02	0.08	0.08
F 4	Cd	Adults	9.83	9.76	9.68	11.59	16.45	11.19	14.74	11.72	9.46	11.80	12.20
		Children	21.66	21.50	21.33	25.54	36.23	24.65	32.47	25.82	20.83	25.98	26.87
	Pb	Adults	3.48	3.32	3.12	6.04	6.70	2.99	6.55	5.11	3.06	5.66	6.07
		Children	7.68	7.31	6.87	13.31	14.77	6.59	14.44	11.25	6.74	12.46	13.38
	Zn	Adults	0.05	0.05	0.04	0.12	0.14	0.04	0.14	0.11	0.04	0.13	0.13
		Children	0.11	0.10	0.10	0.27	0.31	0.08	0.30	0.24	0.09	0.28	0.29
	Cr	Adults	0.01	0.02	0.01	0.04	0.04	0.01	0.04	0.04	0.01	0.04	0.04
		Children	0.03	0.03	0.02	0.08	0.08	0.02	0.08	0.08	0.02	0.08	0.08
F 5	Cd	Adults	10.01	9.91	9.83	12.02	16.88	11.29	14.91	11.95	9.56	11.85	12.27
		Children	22.05	21.83	21.66	26.48	37.18	24.88	32.85	26.32	21.05	26.09	27.04
	Pb	Adults	3.54	3.47	3.26	6.27	7.01	3.15	6.63	5.52	3.18	5.79	6.37
		Children	7.79	7.65	7.17	13.82	15.45	6.93	14.61	12.16	7.01	12.76	14.04
	Zn	Adults	0.05	0.05	0.05	0.13	0.15	0.06	0.15	0.13	0.05	0.12	0.13
		Children	0.12	0.11	0.12	0.29	0.33	0.12	0.32	0.28	0.11	0.27	0.29
	Cr	Adults	0.01	0.01	0.01	0.08	0.08	0.01	0.08	0.08	0.01	0.08	0.08
		Children	0.03	0.02	0.03	0.18	0.18	0.03	0.18	0.18	0.03	0.18	0.18
F 6	Cd	Adults	8.75	8.68	8.60	9.03	11.12	8.80	10.39	8.88	8.63	8.95	9.13
		Children	19.28	19.11	18.95	19.89	24.49	19.39	22.88	19.56	19.00	19.72	20.11
	Pb	Adults	3.01	2.99	2.91	3.39	4.05	2.97	3.54	3.31	2.84	3.17	5.13
		Children	6.63	6.59	6.41	7.47	8.93	6.54	7.80	7.30	6.25	6.98	11.30
	Zn	Adults	0.05	0.04	0.04	0.12	0.13	0.05	0.13	0.11	0.05	0.11	0.11
		Children	0.10	0.10	0.09	0.25	0.29	0.11	0.28	0.24	0.11	0.25	0.25
	Cr	Adults	0.01	0.01	0.01	0.03	0.03	0.01	0.03	0.03	0.01	0.03	0.03
		Children	0.02	0.02	0.03	0.06	0.06	0.02	0.06	0.06	0.02	0.06	0.06
F 7	Cd	Adults	9.66	9.58	9.51	9.73	11.95	9.71	11.02	9.58	9.48	9.66	9.88
		Children	21.27	21.11	20.94	21.44	26.32	21.39	24.27	21.11	20.89	21.27	21.77
	Pb	Adults	3.29	3.28	3.26	3.89	4.69	3.15	4.43	3.46	3.23	3.63	4.21
		Children	7.25	7.22	7.17	8.58	10.34	6.95	9.77	7.61	7.11	7.99	9.28
	Zn	Adults	0.05	0.05	0.05	0.10	0.12	0.04	0.11	0.09	0.04	0.09	0.10
		Children	0.11	0.11	0.10	0.22	0.25	0.09	0.24	0.21	0.09	0.20	0.22
	Cr	Adults	0.01	0.01	0.01	0.05	0.05	0.02	0.05	0.05	0.01	0.05	0.05
		Children	0.03	0.02	0.02	0.10	0.10	0.03	0.10	0.10	0.03	0.10	0.10
F 8	Cd	Adults	9.58	9.38	8.40	11.42	15.54	10.24	14.84	10.69	8.70	10.76	12.22
		Children	21.11	20.67	18.50	25.15	34.24	22.55	32.69	23.55	19.17	23.71	26.93
	Pb	Adults	3.36	3.23	3.08	5.09	6.30	2.94	5.90	4.77	3.03	4.87	5.30
		Children	7.41	7.12	6.79	11.21	13.88	6.47	13.00	10.51	6.66	10.73	11.67
	Zn	Adults	0.04	0.05	0.05	0.11	0.12	0.05	0.11	0.10	0.04	0.10	0.11
		Children	0.09	0.10	0.11	0.23	0.26	0.10	0.24	0.22	0.10	0.22	0.24
	Cr	Adults	0.01	0.01	0.01	0.04	0.04	0.01	0.04	0.04	0.01	0.04	0.04
		Children	0.02	0.03	0.03	0.09	0.09	0.03	0.09	0.09	0.02	0.09	0.09
F 9	Cd	Adults	9.53	9.38	9.43	9.91	12.02	9.73	11.07	9.66	9.56	9.78	9.98
		Children	21.00	20.67	20.78	21.83	26.48	21.44	24.38	21.27	21.05	21.55	22.00
	Pb	Adults	3.39	3.20	3.04	5.06	6.29	2.90	5.87	4.74	2.94	4.84	5.27
		Children	7.47	7.06	6.70	11.14	13.85	6.40	12.93	10.43	6.47	10.67	11.62
	Zn	Adults	0.04	0.05	0.04	0.11	0.13	0.05	0.12	0.11	0.04	0.10	0.10
		Children	0.09	0.11	0.09	0.24	0.29	0.12	0.27	0.23	0.08	0.23	0.22
	Cr	Adults	0.01	0.02	0.01	0.05	0.05	0.01	0.05	0.05	0.01	0.05	0.05
		Children	0.02	0.04	0.02	0.12	0.11	0.03	0.12	0.11	0.01	0.12	0.12
F 10	Cd	Adults	9.73	9.68	9.15	11.54	16.15	10.29	14.56	10.51	9.33	11.49	12.10
		Children	21.44	21.33	20.17	25.43	35.57	22.66	32.08	23.16	20.55	25.32	26.65
	Pb	Adults	3.42	3.26	3.10	5.45	6.29	3.20	6.09	4.79	3.22	5.18	5.84
		Children	7.53	7.17	6.84	12.00	13.85	7.06	13.41	10.56	7.09	11.41	12.87
	Zn	Adults	0.06	0.05	0.05	0.12	0.14	0.04	0.13	0.12	0.05	0.11	0.12
		Children	0.13	0.11	0.10	0.26	0.30	0.09	0.29	0.26	0.11	0.24	0.27
	Cr	Adults	0.01	0.01	0.01	0.03	0.03	0.01	0.03	0.03	0.01	0.03	0.03
		Children	0.02	0.02	0.02	0.07	0.07	0.02	0.07	0.07	0.03	0.07	0.07

The minimum THQ of Pb for adults was 2.84 and the minimum for children was 6.25. These values were connected with *O. gratissimum* source from F6. For maximum values, the respective for adults and children were 7.01 and 37.18, and are connected with *T triangulare* sourced from F5 (Table 4). These values were higher than THQs reported in [18] for some vegetables studied in India; higher than those recorded in Shizhuyuan, China [16]; and higher than values recorded for some vegetables studied in Challawa, Nigeria [41].

The respective minimum Cd THQs for adults and children were 8.32 and 18.34 via *V. amygdalina* sourced from F 2 and 3, while the respective maximum THQs were 16.88 and 37.18 via *T triangulare* sourced from F5 (Table 4). These values exceeded the THQs reported in [18]; in [17]; and in [41] for lettuce ingested by adults and children; tomato and onion ingested by children. Edogbo et al. in [41] reported respective THQ of 32.1 and 8.76 for tomato and onion ingested by adults.

The minimum THQ (0.04) of Zn for adults linked with ingestion of *O. gratissimum* from F1, F3, F4, F7, F8 and F9; *S. macrocarpon* from F1, F4, F7, and F10; *V. amygdalina* from F3, F4, F6 and F9; *T. officinale* from F2 and F6; and *C. olitorius* from F8 and F9. The minimum of 0.08 for children was connected with *S. macrocarpon* and *O. gratissimum* from F4 and F9 respectively. The maximum of 0.13 for adults was connected with *T. occidentalis* from F4; *A. hybridus* and *C. argentae* from F5; *C. rubens* from F4 and F5; and *T triangulare* from F6 and F9. The maximum (0.29) for children was connected with *A. hybridus* from F4; *C. rubens* from F4 and F5; and *T triangulare* from F6 and F9. These values were less than the values reported for leafy vegetables but higher than the values for lettuce taken in by adults and children; and tomato and onion for adults [18]. The study documented in [18] showed respective values of 0.14 and 0.12 for ingested tomato and onion by children – these values are higher than the minimum values for adults and children in this study. Zhou *et al.* in [17] reported a range of 0.003–0.308 for adults and 0.004–0.405 for children.

The minimum THQ (0.01) of Cr for adults was connected with *C. olitorius, T. officinale, V. amygdalina, S. macrocarpon* and *O. gratissimum* from F1, F3, F5, F6 and F10; *C. olitorius, V. amygdalina, S. macrocarpon* and *O. gratissimum* from F4 and F9; *C. olitorius, T. officinale, S. macrocarpon* and *O. gratissimum* from F2; *C. olitorius, T. officinale, V. amygdalina*, and *O. gratissimum* from F7; and *C. olitorius, T. officinale, V. amygdalina and S. macrocarpon* from F8. The minimum (0.01) for children was connected with *O. gratissimum* from F1 and F9.

The maximum THQ (0.08) of Cr for adults was through diet of *T. occidentalis*, *A. hybridus*, *C. argentae*, *C. rubens*, *T triangulare and B alba* from F5. The maximum (0.09) for children was via *T. triangulare* and *B alba* from F3 and F8; and *T. occidentalis*, *A. hybridus*, *C. argentae*, and *C. rubens* from F8.

The THQs calculated for Cd and Pb exceeded one, and the values for Cd were greater than those of Pb. These values are indication of health risks from ingestion of vegetables containing Cd and Pb respectively. The study showed higher health risks for children. This is conspicuously obvious from the higher THQs for children. Zhou et al. in [17] and Edogbo et al. in [41] also reported higher THQs for children. Reason for higher values for children is due to the fact that THQ is affected by RfD and W_AB_ which vary between children and adults [17, 41].

The respective THQs for Zn and Cr for adults and children fell below one-showing no health risk. However, it has been reported that it is difficult to base the health risks of metals on individual values of THQ for the metals [17]. In addition, Wang et al. in [55] stated that exposure to more than one metal may culminate in either additive, or interactive, or both negative health effects. Therefore, the TTHQ which is the summation of THQ of all the metals involved is more feasible for assessing the health risks [17]. On the strength of the calculated TTHQ which was greater than one in this study, Zn and Cr were found to also constitute potential health hazards.

Like the impacts of Cd and Pb, Cr and Zn showed higher health risks for children. The order of constituting health hazard was discovered to be Cd > Pb > Zn > Cr. This implied that Cd has higher adverse impact on human health while Cr has the least health risk in this study. The study in [17] showed metals adverse health impact in the order of Cd > Pb > Zn > Cu > As. Edogbo et al. in [36] discovered THQ >1 for Cd, Cr and Pb except for Zn in their study in Challawa, Nigeria. However, their study discovered (TTHQ >1) for Zn which implied that Cd, Cr Pb and Zn were of potential health concern in Challawa, Nigeria. This current study showed that the use of these vegetables as diet for a long time might cause the children and adults health challenges.

## 4 Conclusion

This study showed that the soils from the farms were polluted with Cd but Cr, Zn and Pb were within their respective safe limits for agro soils; Pb and Cd concentrations in vegetables exceeded the respective MACs (0.3 and 0.1 mg/kg); *T. triangulare* showed the highest BCF for the metals but the lowest were observed in *S. macrocarpon, V. amygdalina*, *O. gratissimum*, and *T. officinale* respectively. The respective HRIs for Cd, Zn and Pb exceeded one for adults and children; the HRIs of Cr exceeded one for children but less than one for adults. For both adults and children, the THQ for Pb, Cd exceeded one but less than one for Zn and Cr; while the TTHQs for the metals exceeded one. HRI >1, THQ >1 and TTHQ >1 indicate health risks in relation to the ingestion of these vegetables, and the health risks were higher for children. The government should ensure that the environmental Laws regulating discharge of mining wastes to soils is strictly enforced in this area, and provide a means of monitoring vegetables supplied from these farms to ensure that they are compliant with health requirements.

## Declarations

### Author contribution statement

Eguakhide Atikpo: Conceived and designed the experiments; Analyzed and interpreted the data; Wrote the paper.

Ehizonomhen Solomon Okonofua: Conceived and designed the experiments; Performed the experiments; Wrote the paper.

Nicholas Omougbo Uwadia: Conceived and designed the experiments; Analyzed and interpreted the data.

Amaka Michael: Analyzed and interpreted the data; Wrote the paper.

### Funding statement

This research did not receive any specific grant from funding agencies in the public, commercial, or not-for-profit sectors.

### Data availability statement

The authors do not have permission to share data.

### Declaration of interests statement

The authors declare no conflict of interest.

### Additional information

No additional information is available for this paper.
